# New insights into the pathogenesis of glucocorticoid-induced avascular necrosis: microarray analysis of gene expression in a rat model

**DOI:** 10.1186/ar3062

**Published:** 2010-06-25

**Authors:** Mohammad Amin Kerachian, Denis Cournoyer, Edward J Harvey, Terry Y Chow, Louis R Bégin, Ayoub Nahal, Chantal Séguin

**Affiliations:** 1 Department of Human Genetics, McGill University Health Center (MUHC), 1650 Cedar Avenue, Montreal, QC H3G 1A4, Canada; 2 Department of Medicine, Division of Haematology, McGill University Health Center (MUHC), 1650 Cedar Avenue, Montreal, QC H3G 1A4, Canada; 3 Department of Oncology, McGill University Health Center (MUHC), 1650 Cedar Avenue, Montreal, QC H3G 1A4, Canada; 4 Division of Orthopaedic Surgery, McGill University Health Center (MUHC), 1650 Cedar Avenue, Montreal, QC H3G 1A4, Canada; 5 Division of Anatomic Pathology, Hôpital du Sacré-Coeur de Montréal, 5400 Gouin Blvd, Montreal, QC H4J 1C5, Canada; 6 Department of Pathology, McGill University Health Center (MUHC), 1650 Cedar Avenue, Montreal, QC H3G 1A4, Canada

## Abstract

**Introduction:**

Avascular necrosis of the femoral head (ANFH) occurs variably after exposure to corticosteroids. Microvascular thrombosis is a common pathological finding. Since systemic thrombophilia is only weakly linked with ANFH, we propose that microvascular vessel pathology may be more related to local endothelial dysfunction and femoral head apoptosis. Corticosteroid effects on the endothelium and resultant apoptosis have been reported. We hypothesize that corticosteroids contribute to a differential gene expression in the femoral head in rats with early ANFH.

**Methods:**

Besides bone marrow necrosis, which is a common sign in ANFH and reported in the early stages, we include the presence of apoptosis in this study as a criterion for diagnosing early disease. Forty Wistar Kyoto (WKY) rats were randomized to either a corticosteroid-treated group or an age-matched control group for six months. After sacrifice, the femoral heads were examined for ANFH. Total mRNA was extracted from femoral heads. Affymetrix exon array (Santa Clara, CA, USA) was performed on 15 selected RNA samples. Validation methods included RT-PCR and immunohistochemistry (IHC).

**Results:**

Although rat exon array demonstrated a significant upregulation of 51 genes (corticosteroid(+)/ANFH(+) *VS *control), *alpha-2-macroglobulin (A2M) *gene was particularly over-expressed. Results were validated by RT-PCR and IHC. Importantly, *A2M *is known to share vascular, osteogenic and cartilage functions relevant for ANFH.

**Conclusions:**

The findings suggest that corticosteroid-induced ANFH in rats might be mediated by *A2M*. Investigation of *A2M *as a potential marker, and a treatment target, for early ANFH should be carried out.

## Introduction

Avascular necrosis of the femoral head (ANFH) is a disabling and progressive condition in young patients, which leads to femoral head collapse and eventual total hip arthroplasty [1,2]. Numerous conditions have been implicated in ANFH [3,4]. Unfortunately, there is currently no biomarker to evaluate the activity status or the prognosis of the disease [5]. The pathogenesis of *idiopathic *ANFH is incompletely understood and therefore predictors of disease initiation or progression are lacking. Two major limitations in the past have impeded the delineation of the pathophysiology: a lack of understanding of the interaction between the disease and the coagulation abnormalities and a lack of suitable animal models. Currently, amongst several pathogenic mechanisms, the *vascular hypothesis*, (or regional endothelial bed dysfunction) in which local microvascular thrombosis leads to a decrease in blood flow in the femoral head [6], has become more accepted. The fact that ANFH is sometimes seen in twins and in familial clusters suggests that genetic factors are also involved [7-10]. New evidence of increased incidence of ANFH in specific animal models provides further evidence of genetic susceptibility [11]. Although observed systemic thrombophilic and hypofibrinolytic coagulation abnormalities in patients with ANFH is increased in some studies compared to controls [12-17], the vast majority of ANFH patients do not demonstrate significant differences in the levels of thrombotic and fibrinolytic factors [18,19].

The current pathophysiological model of ANFH postulates a multiple hit theory such that with an increasing number of risk factors the chance of ANFH increases [20]. Amongst the many risk factors, glucocorticosteroids (GCs) play the leading role in non-traumatic cases of ANFH [21]. Even when GCs are thought to be the cause a careful history is suggested to identify other risk factors. GCs are the mainstay of therapy in most inflammatory disorders and they are also included in most chemotherapy protocols. Therefore, ANFH is thus a potential major complication for large patient populations. Investigators have proposed both direct and indirect effects of GCs on cells. Indirect and direct mechanisms remain intimately related and often result in positive feedback loops to potentiate the disease processes. However, the direct effects, in particularly apoptosis, have recently been shown to be increasingly important. Suppression of osteoblast and osteoclast precursor production, increased apoptosis of osteoblasts and osteocytes, prolongation of the lifespan of osteoclasts and apoptosis of endothelial cells (EC) are all direct effects of GC usage [22]. In the present study, we propose that the microvascular events could be more related to endothelial dysfunction and diffuse femoral head apoptosis. Based on reported data on corticosteroid effects on the endothelium and their role in apoptosis, we hypothesized that corticosteroids contribute to a differential gene expression in rats with early ANFH.

In a previous *in vivo *pilot study, an inbred rat strain susceptible to develop GC-induced ANFH was identified. Here we employed gene profile analysis using this susceptible rat strain in order to study the pathogenesis at an early disease stage. Knowledge of the gene expression pattern and the events that contribute to the genesis and progression of ANFH in this rat model could provide a better understanding of the pathogenesis in humans.

## Materials and methods

### Experimental animals and their maintenance

Forty Wistar Kyoto (WKY) rats (ages four weeks old) were purchased from Charles River Laboratories (Pointe-Claire, QC, Canada). The rats were tagged and housed in plastic cages (two to four animals per cage) under standard laboratory conditions with a 12-hour dark/12-hour light cycle, a constant temperature of 20°C, and humidity of 48%. Food and water were provided *ad libitum *with a standard rodent diet. The weight of the rats was followed before and after the implant of a prednisone pellet for the first three consecutive weeks and then every month until the end of the experiment. All experiments were conducted under an animal protocol approved by the McGill Animal Care Department.

### Glucocorticoid administration

Slow-release prednisone pellets (Innovative Research of America, Sarasota, FL, USA) were implanted subcutaneously into 24 Wistar Kyoto rats (12 males and 12 females rats) at the age of five weeks. Each pellet was implanted beneath the skin on the lateral side of the neck by surgically making an incision and developing a pocket about 2 cm beyond the incision site. The pellet was placed in the pocket and the incision was sutured. Based on the manufacturer's instructions the pellet releases a constant dose of the drug subcutaneously. To maintain a constant dosage during the six-month period of the experiment, second and third pellet implantations were performed using the same procedure at two and three months respectively. The average dose release from the pellet was equivalent to 1.5 mg/kg/day for the period of six months. The dose of corticosteroids and the duration of treatment were chosen based on clinical experience. For the control group, 16 age-matched Wistar Kyoto (eight males and eight females) rats received placebo pellets (Innovative Research of America) introduced through the same surgical technique.

### Histologic examination

The rats were sacrificed with an overdose of ketamine/xylazine at the age of 30 weeks. Tissue samples were obtained from the proximal femur containing the femoral head. Some samples were put in RNALater (QIAGEN Inc., Mississauga, ON, Canada) for RNA extraction and some samples were fixed for histological examination. Bone samples were fixed in 10% neutral buffered formalin, then decalcified in 4% ethylenediamine tetraacetic acid (pH 7.2) (Sigma-Aldrich, St Louis, MO, USA). The specimens were processed routinely and embedded in paraffin. Tissue samples were sectioned parasagitally with a rotary microtome at four to five microns thickness, stained with hematoxylin and eosin and evaluated by light microscopy.

**Figure 1:**
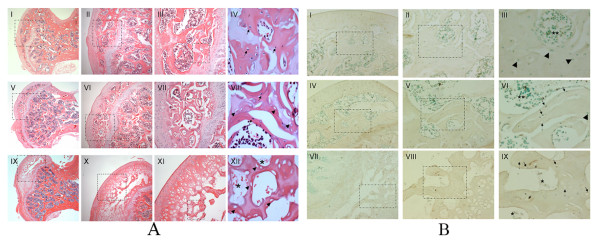
**Histology findings in placebo and steroid-induced ANFH rats**. **(a) **Photomicrographs showing histological findings in placebo- (I-IV) and steroid-treated WKY rats (V-VIII & IX-XII) femoral heads. **I-IV: **No osteonecrosis, normal osteocytes (arrow), **V-VIII**: Early stage of osteonecrosis, normal osteocytes (arrow), empty lacunae (arrow head), **IX-XII**: Late stage of osteonecrosis, empty lacunae (arrow head), complete necrosis of bone marrow (asterisk), H&E staining, **I**, **V**, **IX **×20; **II**, **VI**, **X **×40; **III**, **VII**, **XI **×100; **IV**, **VIII**, **XII **×200, dotted square chosen to be magnified. **(b) **Photomicrographs showing apoptosis of osteocytes as a marker of early ANFH. TUNEL staining apoptosis assay counterstained with 0.5% methyl green solution. **I-III: **Normal femoral head tissues in placebo-treated WKY rats, normal osteocytes (arrow head), normal bone marrow (double asterisk), **IV-VI**: Early stage of osteonecrosis in steroid-treated WKY rats, TUNEL positive osteocytes (arrow), empty lacunae (dotted arrow), normal osteocytes (arrow head), normal bone marrow (double asterisk). **VII-IX**: Late stage of osteonecrosis in steroid-treated WKY rats, TUNEL positive osteocytes (arrow), empty lacunae (dotted arrow), complete necrotic bone marrow (asterisk), **I**, **IV**, **VII **×40; **II**, **V**, **VIII **×100; **III**, **VI**, **IX **×200, dotted square chosen to be magnified.

The tissue samples were analyzed in a blinded fashion by two experienced bone pathologists (AN and LRB). The histological findings of an established ANFH are generally defined as dead trabeculae exhibiting empty lacunae with or without appositional bone formation [23], as shown in Figure 1. While the development of ANFH proceeds through various clinically identifiable stages, it was preferable for this study to detect early as well as late stages of the condition. With this objective in mind, we adopted the criteria of Arlet *et al*, namely degeneration, necrosis, and disappearance of marrow cells as well as the nuclear disappearance and hypochromasia of trabecular osteocytes as early signs of ANFH [24]. Early signs of ANFH were also considered when apoptosis occurred in the osteocytes and osteoblasts (Figure 1). Positivity for apoptosis was defined by the authors as more than three osteocytes and/or osteoblasts recognized in a high magnification field based on previous studies [25,26]. The experiments were performed in triplicate (×200) (Table 1).

**Table 1:** Histological findings of avascular necrosis of the femoral head (ANFH) in Wistar Kyoto rats

Sex	Treatment	No. of rats	OA/GC	OEL	EO	LO
Male	Placebo	6	2	1	1	1
Male	Prednisone	7	5	1	4	1
Female	Placebo	5	1	0	1	0
Female	Prednisone	12	3	2	1	2

The histological findings of an established (late stage) ANFH were defined as empty lacunae. Early signs of ANFH was considered when apoptosis occurred in more than three osteocytes and/or osteoblasts recognized in a high magnification field (×200); EO, number of early stages of osteonecrosis; LO, number of late stages of osteonecrosis; OA/GC, osteocyte apoptosis and/or ghost cell (a denucleated cell with an unstained center where the nucleus has been); OEL, osteocyte empty lacunae.

### Measurement of apoptosis in undecalcified bone section

Terminal dexoynucleotidyl transferase (TdT) mediated deoxyuridine triphosphate biotin nick end labeling (TUNEL) was used to detect fragmented DNA known to be associated with apoptotic cell death. TUNEL assay on paraffin-embedded tissue sections was performed with the DeadEnd Colorimetric TUNEL System (Promega, Madison, WI, USA) as recommended by the manufacturer. Briefly, after deparaffinizing and permibilizing the tissue sections with proteinase K, the slides were incubated with the reaction mixture containing recombinant TdT and biotinylated nucleotide for one hour at 37°C inside a humidified chamber. Labelled DNA was visualized with horseradish-peroxidase-labelled streptavidin using 3,3'-diaminobenzidine (DAB) as the chromogen. DNase I -treated tissue sections were used as positive controls. Negative controls for the study were sample slides processed using the same procedure but not treated with TdT enzyme. All the slides were counterstained with 0.5% methyl green solution (0.5 g ethyl violet (Sigma-Aldrich) in 100 ml sodium acetate buffer, 0.1 M and pH.4.2), cleared, mounted and evaluated by light microscopy.

### RNA extraction from rat bone specimens

Total RNA was extracted by an innovative method consisting of a combination of TRIzol^® ^Reagent (Invitrogen, Carlsbad, CA, USA) and RNeasy Mini kit (QIAGEN Inc.) followed by DNase I treatment (QIAGEN Inc.). Briefly, femoral head specimens were removed from RNALater and washed thoroughly with diethyl pyrocarbonate (DEPC) -treated phosphate buffer solution (PBS). Femoral head specimens were placed in liquid nitrogen. The specimens were ground to a fine powder with a porcelain mortar and pestle. TRIzol^® ^1 ml was then added to each ground femoral head specimen. After vortexing for one minute, the homogenized specimen was incubated for five minutes at room temperature (RT) and 0.2 ml Chloroform (Sigma-Aldrich) was added per 1 ml of TRIzol^®^. After vortex use of 15 seconds the samples were incubated for three minutes at room temperature. The samples were then centrifuged at 12,000 × g for 15 minutes at 4°C. The aqueous phase was removed from each sample and one volume of ethanol was added to it and mixed thoroughly. Up to 700 μl of the sample including any precipitate that may have formed was transferred into an RNeasy Mini Spin Column. The column was then processed according to the RNeasy Mini kit manufacturer instruction. Any Genomic DNA contamination was removed by treating the samples with DNase I. The RNA quality was assessed using RNA 6000 NanoChips with the Agilent 2100 Bioanalyzer (Agilent, Wilmington, DE, USA).

### Affymetrix exon arrays

Affymetrix GeneChip^® ^Rat Exon 1.0 ST array interrogating over 850,000 exon clusters within the known and predicted transcribed regions of the entire genome and about one million probe sets was used. Affymetrix exon array was performed on 15 RNA samples of GC-treated and non-treated rats divided in three groups based on histological evaluation: Group 1- Placebo/ANFH(-); Group 2- GC-treated/ANFH(+) and Group 3- GC-treated/ANFH(-), each group consisting of five samples. Biotin-labelled targets for the microarray experiment were prepared using 1 μg of total RNA. Ribosomal RNA was removed with the RiboMinus Human/Mouse Transcriptome Isolation Kit (Invitrogen, Eugene, Oregon, USA) and cDNA was synthesized using the GeneChip WT (Whole Transcript) Sense Target Labeling and Control Reagents kit as described by the manufacturer (Affymetrix, Santa Clara, CA, USA). The sense cDNA was then fragmented by uracil DNA glycosylase and apurinic/apyrimidic endonuclease-1 and biotin-labeled with terminal deoxynucleotidyl transferase using the GeneChip WT Terminal labeling kit (Affymetrix). Hybridization was performed using five micrograms of biotinylated target, which was incubated with the GeneChip Rat Exon 1.0 ST array (Affymetrix) at 45°C for 16 to 20 h. After hybridization, non-specifically bound material was removed by washing and specifically bound target was detected using the GeneChip Hybridization, Wash and Stain kit, and the GeneChip Fluidics Station 450 (Affymetrix). The arrays were scanned using the GeneChip Scanner 3000 7G (Affymetrix). We used Affymetrix Power tools (Affymetrix), R and in-house built Perl scripts to filter the background noise based on the *detection above background *results that is the detection metric generated by comparing Perfect Match probes to a distribution of background probes. Rat exon array data was analyzed by Dr Daniel Bird from Creative Biomics CD Inc. (Shirley, NY, USA): data were normalized based on the Iter-PLIER algorithm by using Affymetrix Power tools, R and in-house built Perl scripts. The genes with low signal (less than 100) were removed from the study. The differentially expressed genes were detected between three groups (G2 vs G1, G3 vs G2, and G3 vs G1) (*P *< 0.05, Fold Change (FC) > 1.5) using in house built R script, infer with *t*-test and adjusted with *Benjamini and Hochberg FDR *method [27].

### Real-Time Polymerase Chain Reaction (SybrGreen RT-PCR)

Real-time PCR was carried out according to the protocol provided by the manufacturer for the QuantiTect SYBR^®^Green RT-PCR kit (QIAGEN Inc.). QuantiTect Primer Assays (Rn_A2m_1_SG, Rn_Col2a1_1_SG, Rn_Mia1_1_SG, Rn_Actb_1_SG) were provided by QIAGEN Inc and a thermal cycler (Prism 7900, Applied Biosystems, Foster City, CA, USA) was used. The reaction was set up in 10 μl final volume applying the following conditions: cycling 50°C (30 minutes), 95°C (15 minutes) and for 45 cycles the conditions were 94°C (15 sec), 55°C (30 sec) and 72°C (30 sec). For the relative quantification of gene expression, the comparative threshold cycle (ΔCt) method was employed and normalized against β-Actin rRNA, which was measured by the same method. All PCR reactions were performed in triplicate. Control reactions were set up lacking reverse transcriptase to assess the level of contaminating genomic DNA.

### Immunohistochemical (IHC) study

Paraffin-embedded sections were placed at 60°C for 15 minutes, incubated in xylene for 15 minutes, and then transferred sequentially into 100% ethanol, 95% ethanol, 70% ethanol, and 50% ethanol for five minutes at RT. Sections were rinsed in deionised water and the endogenous peroxidase activity was blocked with incubating sections in 3% H_2_O_2 _in distilled water for five minutes. The slides were washed in several changes of distilled water. Antigen was retrieved by incubating the slides in Digest-All™ 3 (Invitrogen Immunodetection, Carlsbad, CA, USA) for 10 minutes. After several washes with PBS the slides were stained using R.T.U. Vectastain^® ^Universal Quick kit (Vector Laboratories, Inc., Burlingame, CA, USA) according to the manufacturer's instructions. Several primary antibodies were used: 1:200 dilution of mouse anti-rat *α-2-macroglobulin *globulin monoclonal antibody (clone 129736, R&D Systems, Minneapolis, MN, USA); prediluted mouse anti-rat *collagen type II alpha 1 *monoclonal antibody (Abcam Inc, Cambridge, MA, USA) or 1:50 dilution of rabbit anti-rat melanoma inhibitory activity (MIA) polyclonal antibody (Santa Cruz Biotechnology, Santa Cruz, CA, USA). According to the manufacturer's instructions the secondary antibody is a prediluted biotinylated antibody manufactured in horse, which recognizes rabbit IgG, mouse IgG and goat IgG. The slides were counterstained with 0.5% methyl green solution as described before.

### Statistical analysis

Data reported on microarray results utilized in-house Perl scripts with *t*-test and adjusted with B-H FDR method to examine differentially expressed genes between two groups (*P *< 0.05, Fold Change (FC) > 1.5). RT-PCR results were given as the mean ± standard error of the mean (SEM). Comparison between groups was made with Student's *t*-test. For small size samples Mann-Whitney U test was used since normal distribution of data was not assumed. Differences were considered significant at *P*-values less than 0.05. Principle component analysis (PCA) was performed using R package to provide a global view of how the various sample groups were related.

## Results

### Histological and apoptosis findings

Histological findings displayed normal, early and late stages of ANFH based on the presence or absence of osteocytes in the lacunaes (Figure 1a). The use of the TUNEL assay to detect apoptosis showed apoptotic osteocytes were located in the osteonecrotic samples without features of inflammation and visible necrosis, such as hyperemia, round cell infiltration, or lipid cyst formation. There was no appositional bone formation associated with granulation tissue around dead bone in keeping with the early stages of ANFH (Figure 1b, IV-X). When the same TUNEL reaction was performed on control tissue (without prior digestion with DNase), a fewer number of cells (one or two) were labeled (Figure 1b, I-III).

### Microarray analysis

In the Affymetrix analysis, G2 replicates were compared with G1 and G3, separately, and G3 replicates were compared with G1 to generate a list of differentially expressed genes. The results were analyzed by a defined set of criteria in which the altered expression of a gene must have at least a change of ± 1.5-fold (FC = fold change) and a *P*-value less than 0.05. These criteria resulted in the identification of 51 genes with significant modulation in G2 compared with G1 and six genes with significant modulation for G3 to G2 (Tables 2 and 3). They also identified 229 genes in G3 versus G1 (Table 4). In this table, only the genes with a change of ± 1.8-fold (FC = fold change) are represented due to the exhaustive list of genes. Although rat exon array demonstrated a significant upregulation of 51 genes when comparing G2 to G1, *alpha-2-macroglobulin *gene was particularly found to be overexpressed when comparing steroid-treated Wistar Kyoto rats which had developed ANFH (G2) to placebo rats (G1) (FC = 3.52, *P *= 0.0005). *Collagen type II alpha-1 (Col2A1) *and *Melanoma Inhibitory Activity-1 (MIA) *genes were also found to be significantly overexpressed by exon array analysis (FC = 2.52, *P *= 0.0005 and FC = 2.29, *P *= 0.0008 respectively). The downregulation of some genes was not considered significant in terms of fold change compared to the upregulated genes; therefore we were able to focus on the genes that were upregulated. Significantly modulated genes were categorized into clusters according to their biological functions using DAVID, a functional annotation tool provided by National Institute of Allergy and Infectious Diseases-NIH. Modulated genes were grouped mainly into clusters of skeletal development, ossification and bone remodelling. Other functional classes significantly represented in the steroid-induced avascular necrosis included response to steroid stimulus response, apoptosis, blood vessel morphogenesis, vasculature development, cell growth, proliferation and differentiation associated genes. In comparison of G3 versus G1, *A2M *and *Col2A1 *were not significantly overexpressed whereas *MIA *was found to be the most up-regulated gene in that group comparison (FC = 3.71, *P *= 0.00).

**Table 2:** Differentially expressed genes from comparing Group 2 (G2) versus Group 1 (G1)

Annotation		PV	FC
NM_012488	alpha-2-macroglobulin	0.0005	+ 3.52
NM_012929	collagen, type II, alpha 1	0.0005	+ 2.52
NM_030852	melanoma inhibitory activity 1	0.0008	+ 2.29
NM_033499	scrapie responsive gene 1	0.0054	+ 2.08
NM_017094	growth hormone receptor	0.0142	+ 1.93
NM_053669	SH2B adaptor protein 2	0.0213	+ 1.89
NM_080698	fibromodulin	0.0099	+ 1.87
NM_133523	matrix metallopeptidase 3	0.0034	+ 1.87
NM_012999	Proprotein convertase subtilisin/kexin type 6	0.0117	+ 1.80
NM_138889	cadherin 13	0.0049	+ 1.77
NM_031808	calpain 6	0.0086	+ 1.73
NM_001002826	murinoglobulin 2	0.0022	+ 1.72
NM_145776	solute carrier family 38, member 3	0.0040	+ 1.71
NM_012846	fibroblast growth factor 1	0.0441	+ 1.70
NM_017058	vitamin D receptor	0.0065	+ 1.69
NM_001009662	carbonic anhydrase 8	0.0275	+ 1.68
NM_031590	WNT1 inducible signaling pathway protein 2	0.0105	+ 1.67
NM_012587	integrin binding sialoprotein	0.0276	+ 1.66
NM_053816	calcitonin receptor	0.0316	+ 1.63
NM_013191	S100 protein, beta polypeptide, neural	0.0123	+ 1.62
NM_031828	potassium large conductance calcium-activated channel, subfamily M, alpha member 1	0.0018	+ 1.62
NM_133569	angiopoietin-like 2	0	+ 1.62
NM_199398	pannexin 3	0.0032	+ 1.62
NM_053605	sphingomyelin phosphodiesterase 3, neutral	0.0126	+ 1.62
NM_170668	solute carrier family 13 (sodium-dependent citrate transporter), member 5	0.0167	+ 1.60
NM_053977	cadherin 17	0.0233	+ 1.60
NM_199407	unc-5 homolog C (C. elegans)	0.0002	+ 1.60
NM_012620	serine (or cysteine) peptidase inhibitor, clade E, member 1 (also designated plasminogen activator inhibitor-1 or PAI-1)	0.0003	+ 1.60
NM_022667	solute carrier organic anion transporter family, member 2a1	0.0055	+ 1.59
NM_001034009	melanoma cell adhesion molecule	0.0032	+ 1.58
NM_053288	orosomucoid 1	0.0236	+ 1.57
NM_031131	transforming growth factor, beta 2	0.0015	+ 1.57
NM_013059	alkaline phosphatase, liver/bone/kidney	0.0218	+ 1.57
NM_133303	basic helix-loop-helix domain containing, class B3	0.0114	+ 1.56
NM_198768	immunoglobulin superfamily, member 10	0.0467	+ 1.55
NM_001017479	transmembrane protein 100	0.0431	+ 1.54
NM_020073	parathyroid hormone receptor 1	0.0370	+ 1.54
NM_024400	a disintegrin-like and metallopeptidase (reprolysin type) with thrombospondin type 1 motif, 1	0.0086	+ 1.54
NM_001014043	sphingomyelin synthase 2	0.0131	+ 1.53
NM_023970	transient receptor potential cation channel, subfamily V, member 4	0.0219	+ 1.52
NM_020656	parvin, alpha	0.0072	+ 1.52
NM_175578	regulator of calcineurin 2	0.0390	+ 1.52
NM_031655	latexin	0.0080	+ 1.52
NM_001013218	receptor accessory protein 6	0.0045	+ 1.52
NM_001005562	cAMP responsive element binding protein 3-like 1	0.0376	+ 1.50
NM_001017496	chemokine (C-X-C motif) ligand 13	0.0140	- 0.55
ENSRNOT00000060250	similar to T-cell receptor alpha chain precursor V and C regions (TRA29)	0.0154	- 0.64
NM_203410	interferon, alpha-inducible protein 27-like	0.0325	- 0.64
NM_001008836	RT1-CE13//RT1 class I, CE13	0.0157	- 0.64
NM_001002280	MAS-related GPR, member X2	0.0021	- 0.66
NM_001008855	RT1 class Ib gene, H2-TL-like, grc region (N3)	0.0350	- 0.67

(+), positive regulation, (-), negative regulation; FC, fold change; V, *P-v*alue.

**Table 3:** Differentially expressed genes from comparing group 3 (G3) versus 2 (G2)

Annotation		PV	FC
NM_001012357	chemokine (C-C motif) ligand 9	0.0371	+ 1.86
NM_013153	hyaluronan synthase 2	0.0103	+ 1.70
NM_030852	melanoma inhibitory activity 1	0.0082	+ 1.62
NM_001012072	protein phosphatase 1, regulatory (inhibitor) subunit 3C	0.0411	+ 1.58
NM_001009639	tubulin polymerization-promoting protein family member 3	0.0243	+ 1.56
NM_012497	aldolase C	0.0155	+ 1.54

(+), positive regulation; (-), negative regulation; FC, fold change; PV, *P*-value.

**Table 4:** Differentially expressed genes from comparing Group 3 (G3) versus 1 (G1). Only genes with fold change above 1.8 have been shown

Annotation		PV	FC
NM_030852	melanoma inhibitory activity 1	0.0000	+ 3.71
NM_001002826	murinoglobulin 2	0.0104	+ 2.64
NM_031808	calpain 6	0.0002	+ 2.62
NM_019189	hyaluronan and proteoglycan link protein 1	0.0000	+ 2.61
NM_001002826	murinoglobulin 2	0.0007	+ 2.37
NM_001012034	ADP-ribosyltransferase 3	0.0015	+ 2.36
NM_057104	ectonucleotide pyrophosphatase/phosphodiesterase 2	0.0091	+ 2.25
NM_001009662	carbonic anhydrase 8	0.0010	+ 2.24
NM_013191	S100 protein, beta polypeptide, neural	0.0002	+ 2.24
NM_138898	phospholipase B	0.0165	+ 2.18
NM_134432	angiotensinogen (serpin peptidase inhibitor, clade A, member 8)	0.0294	+ 2.16
NM_012620	serine (or cysteine) peptidase inhibitor, clade E, member 1	0.0036	+ 2.15
NM_031828	potassium large conductance calcium-activated channel, subfamily M, alpha 1	0.0003	+ 2.15
NM_133523	matrix metallopeptidase 3	0.0162	+ 2.15
NM_138889	cadherin 13	0.0011	+ 2.14
NM_133569	angiopoietin-like 2	0.0000	+ 2.14
NM_001012163	LIM and senescent cell antigen like domains 2	0.0048	+ 2.13
NM_001013213	integrin beta 3 binding protein (beta3-endonexin)	0.0013	+ 2.10
NM_198748	scinderin	0.0017	+ 2.09
NM_012497	aldolase C	0.0002	+ 2.08
NM_031694	heat shock factor 2	0.0085	+ 2.05
NM_198768	immunoglobulin superfamily, member 10	0.0015	+ 2.03
NM_053977	cadherin 17	0.0062	+ 2.02
NM_001014060	similar to SRY (sex determining region Y)-box 5 isoform a	0.0002	+ 1.97
NM_012999	proprotein convertase subtilisin/kexin type 6	0.0025	+ 1.96
NM_013080	protein tyrosine phosphatase, receptor-type, Z polypeptide 1	0.0072	+ 1.94
NM_001002819	glutamine-fructose-6-phosphate transaminase 2	0.0096	+ 1.93
BC079425	hypothetical protein LOC654482	0.0009	+ 1.92
NM_031131	transforming growth factor, beta 2	0.0013	+ 1.91
NM_022927	midline 1	0.0070	+ 1.90
NM_181366	G protein-coupled receptor 64	0.0010	+ 1.90
NM_022230	stanniocalcin 2	0.0003	+ 1.89
NM_199398	pannexin 3	0.0021	+ 1.87
NM_053605	sphingomyelin phosphodiesterase 3, neutral	0.0053	+ 1.86
NM_001009647	mitochondrial ribosomal protein L16	0.0008	+ 1.85
NM_001077641	phospholipase C, beta 1	0.0116	+ 1.85
NM_020073	parathyroid hormone receptor 1	0.0016	+ 1.83
NM_017135	adenylate kinase 3-like 1	0.0149	+ 1.83
NM_013000	peptidylglycine alpha-amidating monooxygenase	0.0065	+ 1.82
NM_001007656	microtubule-associated protein, RP/EB family, member 3	0.0008	+ 1.81
NM_031590	WNT1 inducible signaling pathway protein 2	0.0002	+ 1.81
NM_022382	phosphodiesterase 4D interacting protein (myomegalin)	0.0127	+ 1.80
NM_134327	CD69 antigen	0.0141	- 0.65
NM_019295	CD5 antigen	0.0114	- 0.65
NM_013121	CD28 antigen	0.0136	- 0.65
NM_031147	cold inducible RNA binding protein	0.0025	- 0.64
NM_001012226	signal transducer and activator of transcription 4	0.0246	- 0.63
NM_001008855	RT1 class Ib gene, H2-TL-like, grc region (N3)	0.0005	- 0.60
NM_001012461	deoxynucleotidyltransferase, terminal	0.0140	- 0.59
NM_173096	myxovirus (influenza virus) resistance 1	0.0186	- 0.59
NM_001009680	2 ' -5 ' oligoadenylate synthetase 1I	0.0039	- 0.58
NM_001008836	RT1 class I, CE13	0.0111	- 0.56
NM_203410	interferon, alpha-inducible protein 27-like	0.0018	- 0.51

(+), positive regulation, (-), negative regulation; FC, fold change; PV, *P*-value.

### Real time PCR Verification of GeneChip Data

From the microarray results, the three genes *(α-2-macroglobulin (A2M), collagen type II alpha-1 (Col2A1), melanoma inhibitory activity-1 (MIA)) *showing the highest upregulation or fold change were selected for validation by means of RT-PCR. The directional fold change was confirmed for all three genes and the correlation with microarray results was established. Some variations, however, were noted in the fold-change values demonstrated by real time PCR compared with values obtained by GeneChip analysis (for *A2M*, FC = 3.52 with exon array and 5.85 with RT-PCR). Variations in fold change values between GeneChip and real time PCR might have been due to different methods of normalization and specificity/sensitivity of each method but the trends were the same for the two methods (differences with *P-*values: 0.005 to 0.0009, Table 5).

**Table 5:** Correlation of gene expression comparing Groups 2 (G2) and 1 (G1) as assessed by microarray and real time PCR (*P *< 0.005 for all genes)

Annotation		Fold Change of a signal	
		
		Microarray	Real time PCR
NM_012488	alpha-2-macroglobulin	3.52	5.85
NM_012929	collagen, type II, alpha 1	2.52	4.42
NM_030852	melanoma inhibitory activity 1	2.29	2.80

### Immunohistochemistry

**Figure 2:**
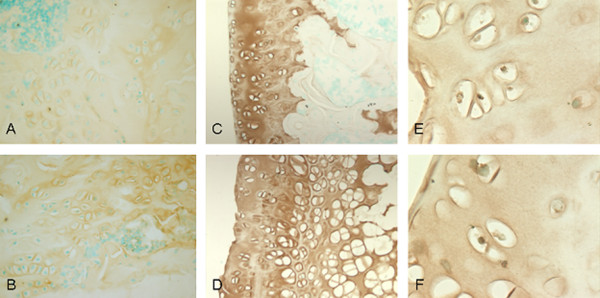
**Upregulation of A2M surface protein expression in steroid-induced early ANFH**. Immunohistochemistry comparing the A2M **(a, b)**, COL2A1 **(c, d)** and MIA (CD RAP) **(e, f)** protein expression between G1 (a, c, e) and G2 (b, d, f) WKY rats, showing enhancement of A2M expression in G2 compared to G1 but no enhancement shown for COL2A1 and MIA genes; brown color demonstrates protein expression and green color displays intact nucleus of cells, (a-d) ×40, (e, f) ×100.

We performed immunohistochemistry staining on the three candidate genes which showed the highest upregulation, *A2M, Col2A1 and MIA*, when comparing G2 to G1. Protein expression of *A2M *was shown to be increased in rats induced with steroids and developing ANFH (Group 2) as compared to the placebo rats without ANFH (Group 1) thus correlating with the mRNA expression levels from GeneChip analysis and RT-PCR method (Figure 2). Notably, immunohistochemical findings for the two other genes of interest (*COL2A1 and MIA*) failed to show enhanced protein expression.

## Discussion

The early events in the pathogenesis of ANFH are incompletely understood due to a typically late diagnosis after fracture and collapse of the femoral head. Besides bone marrow changes, evidence has shown that apoptosis is involved in the early stages of steroid-induced osteonecrosis [26]. Weinstein *et al. *reported that the number of apoptotic bone cells increased significantly in mice after steroid administration [28]. Recent studies have shown apoptotic cells in clinical and animal models of GC-induced ANFH [26,29,30].

In previous studies, we characterized an inbred rat (WKY) susceptible to develop steroid-induced osteonecrosis [31]. It is possible that this strain of rats has genetically predisposing factors to develop ANFH and additional risk exposures (GC) will facilitate the development of the disease. In our animal model, prednisone administration enhanced the incidence of the disease in up to 75% (6/8) of the male WKY rats, suggesting it is a suitable model. In the literature, 5 to 15 week-old rats have been used to study non-traumatic ANFH [23,26,32]. In the current study, WKY rats started to receive continuous steroid dosage released from the pellets at the age of five weeks for 25 weeks. Harvest at six months showed classical histological signs of early ANFH.

For the Affymetrix GeneChip findings, comparison of G2 versus G1 indicated that multiple pathological reactions occurred. According to the functional annotation tool (DAVID), modulated genes in the comparison of G2 and G1 (Table 2) were grouped mainly into skeletal development, ossification and bone remodelling. Functional clusters of genes were significantly represented by steroid stimulus response, apoptosis, blood vessel morphogenesis, vasculature development, coagulation-related, cell growth, proliferation and differentiation associated genes.

The expression of steroid stimulus response genes (*A2M, alkaline phosphatase, tissue-nonspecific, transforming growth factor beta 2 *and *potassium large conductance calcium-activated channel, subfamily m, alpha member 1*) were, as predicted, altered significantly. Previous *in vivo *and *in vitro *models as well as clinical studies showed that steroids induce apoptosis in osteoblasts and osteocytes [30,33-35]. Amongst the 51 differentially regulated genes identified in our gene array analysis (Table 2), five genes (*S100 protein-beta polypeptide, transforming growth factor-beta 2, vitamin D receptor, unc-5 homolog c (C. elegans) and growth hormone receptor*) are in fact components of the apoptosis pathway.

The process of apoptosis can be directly induced by steroids but is also related to thrombosis in the blood vessels of the femoral head. In fact, the vascular hypothesis (regional endothelial bed dysfunction) appears to be relevant in the pathogenesis of ANFH. Damage or activation of femoral head endothelial cells results in abnormal blood coagulation and thrombi formation [36]. Due to heterogeneity of the phenotype expression between endothelial cells in the body, a local endothelial cell dysfunction can occur where the femoral head endothelial cells react differently to the ANFH risk factors (GCs) than other endothelial cells in the body. In keeping with the theory of endothelial cell activation having a role in ANFH, coagulation-related gene expression in particular *serine (or cysteine) peptidase inhibitor, clade E, member 1 *also named *plasminogen activator inhibitor 1 (PAI-1)*, a serine protease inhibitor that is synthesized and released by endothelial cells in the blood, was shown to be significantly over-expressed in this study. An increase in *PAI-1 *suppresses the generation of plasmin resulting in hypofibrinolysis and a relative hypercoagulable state [1]. Decreased fibrinolytic activity, which may be a consequence of increased *PAI-1*, has been described in patients with ANFH [37], although a few studies have reported that there were no significant differences in the levels of thrombotic and fibrinolytic factors [18,19].

Similarly, our findings demonstrate that several genes involved in the dynamic remodelling structure of the femoral head are also shown to be differentially expressed in ANFH (Table 2). Clinically this may be relevant in that if the balance between degradation and repair (bone remodelling) becomes shifted to degradation and bone loss by the effect of GC, a failure of structural integrity at the subchondral region of bone with collapse could occur.

In the present study, results showed *A2M *gene expression to be the most significantly upregulated gene when comparing G2 to G1. Correlation was obtained at the microarray, RT-PCR as well as the protein level as demonstrated by IHC study results. Most importantly, *A2M *was not significantly upregulated when comparing G3 to G1. *A2M *is a plasma-derived matrix metalloproteinase inhibitor which obstructs cartilage degradation induced by matrix metalloproteinases [38]. The literature supports the role of corticosteroids in the modulation of *A2M *[39,40]. In both reports, corticosteroids were shown to enhance *A2M *levels. *A2M *is reported as being implicated in cartilage degradation [41], and as an osteogenic growth peptide (OGP) - binding protein. Activated *A2M *may thus participate in the removal of OGP from the system [42]. Additional reports suggest inhibition of BMP-1 (bone morphogenic protein-1) by *A2M *[43]. *A2M *has been identified on the luminal surface of endothelial cells in sections of normal human arteries and veins [44]. *A2M *has also been implicated in hemostasis as a regulator of thrombin [45] and in the development of thromboembolism in children [46]. Together, all these findings suggest that *A2M *shares haemostatic, cartilaginous and osteogenic properties and may have a potential role in the development of early steroid-induced ANFH. Determination of whether *A2M *over-expression in our study is either the result or the cause of the apoptosis found in our rats developing early ANFH following administration of steroids, will require further study.

Two other genes of interest, *Col2A1 *and *MIA*, were also shown to be over-expressed significantly by microarray analysis and RT-PCR results but immunohistochemical study failed to show an increased cell surface expression of these genes.

Comparing the gene profiling of G3 versus G2, six genes stood out in our analyses (Table 3). Although G3 animals have not developed ANFH, their gene profile reflects inhibition of osteoblast proliferation, differentiation and osteoclast activation. Perhaps most osteogenic cells in this group have not gone through the apoptotic phase and there are more viable cells expressing these molecules in comparison to G2. Differences could also be explained in that gene expression analysis findings are supportive of a result effect indicating steroid treatment and a disease effect affecting the apoptotic process are involved in the early stages of ANFH. Secondly, a genetic variation based on differences in transcription and translation could provide an explanation for the phenotypic differences found in our study. Thirdly, epigenetic variation, resulting from the interaction between the genotype and the environment, is also a potential process that could explain the findings that not all treated animals developed early ANFH when submitted to the same experimental conditions. Also, any of the genes listed in the comparison of G3 to G2 (Table 3) with the exception of *MIA*, could have a protective effect against the development of steroid-induced early AVN. Similarly, the absence of *A2M *over-expression in that same group comparison G3 to G2, and in group comparison G3 to G1 is consistent with the phenotypic absence of early ANFH in rats representing G3.

## Conclusions

In summary, it is postulated that multiple pathological reactions occur during ANFH. Genetic predisposition contributes to the development of ANFH. There is normally a balance between degenerative and regenerative molecules in the bone environment of the femoral head. GCs may trigger a degenerative process as well as inhibit the repair. In this study, several molecules are significantly upregulated and could be involved in the pathogenesis of ANFH. However, only *A2M *gene over-expression has been consistently found at the microarray, RT-PCR and protein level for the three genes showing the most significant upregulation. Besides, *A2M *was not significantly upregulated in rats administered steroids but without developing the disease. Thus, *A2M *seems to be a possible biomarker more of ANFH itself (induced by steroids) than a marker of steroids alone. It remains to be determined in which specific pathway (although likely in the endothelial cell activation and/or the apoptosis pathway) and at which level, the effect of this gene occurs in corticosteroid-induced ANFH. Identifying its role within a specific pathway will likely lead to a better understanding of the molecular events that follow the administration of corticosteroids and subsequent irreversible necrosis and bone collapse. Obviously, investigation of the use of *A2M *as a potential marker for the early warning of ANFH should be carried out.

ArrayExpress accession code: [E-MEXP-2751].

## Abbreviations

A2M: alpha-2-macroglobulin; ANFH: avascular necrosis of the femoral head; BMP-1: bone morphogenic protein-1; Col2A1: collagen type II alpha-1; ΔCt: comparative threshold cycle; DAB: 3:3'-diaminobenzidine; DEPC: diethyl pyrocarbonate; EC: endothelial cells; FC: fold change; G1: group 1; G2: group 2; G3: group 3; GCs: glucocorticosteroids; IHC: immunohistochemistry; MIA: Melanoma Inhibitory Activity-1; OGP: osteogenic growth peptide; PAI-1: plasminogen activator inhibitor 1; PCA: principle component analysis; PBS: phosphate buffer solution; RT: room temperature; RT-PCR: real-time polymerase chain reaction; SEM: standard error of the mean; TDT: terminal dexoynucleotidyl transferase; TUNEL: terminal dexoynucleotidyl transferase mediated deoxyuridine triphosphate biotin nick end labelling; WKY: Wistar Kyoto.

## Competing interests

CS has applied for a provisional patent for A2M (α-2-Macroglobulin) as a diagnostic assay for Avascular Necrosis of the Femoral Head. The other authors declare that they have no competing interests.

## Authors' contributions

All authors participated in the study. MAK made a major contribution to the writing of the manuscript's first draft, and conducted the experiments involved in the study. CS made a major contribution to the design of the study, data interpretation and scientific revision of the manuscript. DC, EJH and TYC made equal contributions to data interpretation and scientific revision of the manuscript. EJH made a major contribution to the editing and grammar of the manuscript. LRB and AN made major contributions to the histological experiments involved in the study. All authors participated in the manuscript preparation and revision. All authors read and approved the final manuscript.
